# A Loosely Coupled Extended Kalman Filter Algorithm for Agricultural Scene-Based Multi-Sensor Fusion

**DOI:** 10.3389/fpls.2022.849260

**Published:** 2022-04-25

**Authors:** Meibo Lv, Hairui Wei, Xinyu Fu, Wuwei Wang, Daming Zhou

**Affiliations:** School of Astronautics NPU, Northwestern Polytechnical University, Xi’an, China

**Keywords:** loosely coupling, extended Kalman filter algorithm, multi-sensor fusion, robustness, agricultural robot

## Abstract

With the arrival of aging society and the development of modern agriculture, the use of agricultural robots for large-scale agricultural production activities will become a major trend in the future. Therefore, it is necessary to develop suitable robots and autonomous navigation technology for agricultural production. However, there is still a problem of external noise and other factors causing the failure of the navigation system. To solve this problem, we propose an agricultural scene-based multi-sensor fusion method *via* a loosely coupled extended Kalman filter algorithm to reduce interference from external environment. Specifically, the proposed method fuses inertial measurement unit (IMU), robot odometer (ODOM), global navigation and positioning system (GPS), and visual inertial odometry (VIO), and uses visualization tools to simulate and analyze the robot trajectory and error. In experiments, we verify the high accuracy and the robustness of the proposed algorithm when sensors fail. The experimental results show that the proposed algorithm has better accuracy and robustness on the agricultural dataset than other algorithms.

## Introduction

In recent years, with the development of artificial intelligence technology, agricultural robots such as drones and ground mobile carts (Katsigiannis et al., 2016; Tang et al., 2020; Atefi et al., 2021; Qin et al., 2021) have been gradually applied to modern agriculture. Their ability to sense the environment and navigate on their own is a more critical influencing factor. And multi-sensor fusion technology provides an effective method for agricultural robots to enhance their ability to work in complex and uncertain environments (Noguchi et al., 1998; Viacheslav et al., 2011).

Multi-sensor fusion technology is a multi-level complementary. It optimally processes the information from different types of sensors to form a reasonable interpretation of the observed environment. Compared with the traditional single-sensor technology, it is fault-tolerant, complementary, real-time, economical, and can solve the defects caused by single sensor, such as fuzzy points and so on. And all of these allow it a more accurate observation of the environment. Therefore, multi-sensor fusion technology has received wide attention in various fields such as military, control, and signal processing (Abidi and González, 1992; Hall and Llinas, 1997; Varshney, 2000).

Visual inertial odometry (VIO) is an application of multi-sensor fusion technology. At present, the mainstream VIOs includes VINS_MONO (Tong et al., 2018), VINS_FUSION (Tong et al., 2018) and MSCKF_VIO (Mourikis and Roumeliotis, 2007). They are used to accomplish map construction, navigation and positioning functions by fusing visual sensors and inertial measurement units (IMUs). According to the difference of fusion framework, vision inertial odometry can be further divided into two types: tightly coupled and loosely coupled. In the loosely coupled (Faessler et al., 2015, 2016; Delmerico and Scaramuzza, 2018), the visual motion and inertial navigation system has two independent modules. In addition, the filter is used to decouple and fuse the visual and IMU information, which has the characteristic of simplicity and speed. VINS-MONO is an open source VIO algorithm, which is realized by tight coupling method and restores the scale through Monocular and IMU. VINS-FUSION is an optimization-based multi-sensor state estimator, it achieves accurate self-localization for autonomous applications (drones, cars, and AR/VR). VINS-FUSION is an extension of VINS-MONO, which supports multiple visual-inertial sensor types (MONO camera + IMU, stereo cameras + IMU, even stereo cameras only). MSCKF_VIO is a binocular visual odometry based on multi-state constraint Kalman filter. Multi-state constraint refers to adding the camera pose of multi-frame images to the Kalman state vector by using least square optimization and estimating 53 the spatial position of feature points through the constraints between multi frame images. Then the state vector is constrained based on the spatial position of the optimized feature points. In the field of agricultural robots, the research on machine vision and trajectory navigation is gradually deepening, and visual-inertial navigation combined with other methods is constantly evolving.

The core of VIO algorithm is based on the state optimization of filtering methods (Scaramuzza et al., 2014), where the filtering methods are mainly based on Bayesian estimation theory, including Kalman filter (KF) algorithm (Gao et al., 2017) and particle filtering algorithm (Leutenegger et al., 2015), etc. Among these, the KF algorithm is used more widely in practical applications. Since SLAM methods are generally non-linear when performing system observations and measurements, KF algorithms need to be extended to the non-linear domain. Accordingly, researchers propose the Extended Kalman Filter (EKF) (Kalman, 1960), a linear approximation method in ignoring higher-order terms for non-linearity, which can estimate the state of a dynamic system from a series of measurements that do not exactly contain noise. Though it is a suboptimal filtering algorithm, it solves the problem of nonlinear systems in the KF algorithm. EKF is widely used in the field of robotics. An improved covariance Intersection EKF data fusion algorithm is proposed for multi-sensor time-delay system (Lee and Gao, 2019). A slam method with extended Kalman filter (EKF) is introduced to locate landmark robots and mobile robots (Inam et al., 2020).

Many studies on multi-sensor fusion algorithms for agricultural robots have been conducted by reasearchers. At the end of last century, many scholars proposed vision based automatic agricultural machine perception, navigation obstacle avoidance and other related methods (Ollis and Stentz, 1997; Sharma and Borse, 2016; Reina and Messina, 2019). Recently, research has developed rapidly, including developed modular structured robots that use GPS for navigation and positioning, and multi-sensor fusion for robot obstacle avoidance (Liu et al., 2011). A multi-sensor data fusion algorithm has been presented based on the fusion set, it is mainly used for data collection in agricultural systems (Hu and Yan, 2018). A multi-sensor fusion approach has been developed for autonomous navigation of agricultural vehicles, which is applied for crop row tracking and traversable operations (Benet and Lenain, 2017). Along with the great improvement in integrated navigation and sensor fusion, a class of autonomous driving control algorithms has been proposed in order to achieve high-precision autonomous navigation of tracked agricultural vehicles, which includes GNSS-RTK sensor integration algorithm and path tracking algorithm (Han et al., 2020). A LiDAR-based autonomous navigation system is developed for agricultural robots, which fuses LiDAR and IMU to solve the problem of agricultural navigation when the tree canopy is obscured (Velasquez et al., 2021). At present, the research of agricultural robots combined with multi-sensor fusion technology is in a rapid development stage. However, the sensor technology generally relies too heavily on GNSS or GPS navigation system, and the sensor fusion category is single, generally using only two sensors for fusion, which may lead to a failure of the whole system when the navigation system has problems.

This paper presents a multi-sensor fusion algorithm based on a loosely coupled extended Kalman filter, the proposed method reincorporates the robot odometer (ODOM), global navigation and positioning system (GPS), and the inertial measurement unit (IMU) on the top of the visual odometer for agricultural robots. And due to the favorable features of GPS navigation such as wide coverage, strong resistance to climate influence and real-time dynamics (RTK), we introduce a loosely coupled and extended Kalman filtering algorithm to fuse the GPS and VINS-MSCKF, ODOM, and IMU. In addition, the effects of GPS or sensors failure on the system are also analyzed. Based on the analysis, it is obvious that the proposed algorithm can better solve the problem for the system downtime situation caused by the failures of GPS and VIO sensors. Based on these experimental results, we can conclude that the proposed algorithm can effectively improve navigation accuracy and system robustness under agricultural scenes.

## Extended Kalman Filtering and Multi-Sensor Fusion Review

### Extended Kalman Filter

As a linearized approximation method, extended Kalman filtering (Sastry, 1971) is a class of extended form of standard Kalman filtering in nonlinear systems.


(1.1) $$\left\{ \begin{array}{c} x_{k+1}=f\,\left(x_{k}\right)+w_{k}\\ z_{k}=h\,\left(x_{k}\right)+v_{k} \end{array}\right.$$

where *x_k_* and *z_k_* are the state vector and the measurement vector, *w_k_* and *v_k_* are system noise and measurement noise, respectively, with covariance *Q_k_*,*R_k_*. The system state equation is:


(1.2) $$x_{k+1}=f\,\left({\hat{x}}_{k\vee k}\right)+F_{k}\,\left(x_{k}-{\hat{x}}_{k\vee k}\right)+w_{k}$$

where, $F_{k}={\partial f/\partial x_{k}|}_{x_{k}={\hat{x}}_{k|k}}$

One-step state prediction equation:


(1.3) $${\hat{x}}_{k+1\vee k}=E\,\left[x_{k+1}+w_{k}\right]=f\,\left({\hat{x}}_{k+|k}\right)$$

The one-step state prediction covariance is:


(1.4) $$P_{k+1\vee k}=F_{k}\,P_{k|k}\,F_{k}^{T}+Q_{k}$$

One-step measurement prediction equation:


(1.5) $${\hat{z}}_{k+1|k}=E\,\left[h_{k+1}+v_{k}\right]=h\,\left({\hat{x}}_{k+1|k}\right)$$

Measurement prediction error covariance array:


(1.6) $$P_{z\,z,k+1|k}=H_{k+1}\,P_{k+1|k}\,H_{k+1}^{T}+R_{k+1}$$

The reciprocal covariance matrix between the state and the measurement equations:


(1.7) $$P_{x\,z,k+1|k}=P_{k+1|k}\,H_{k+1}^{T}$$

State gain matrix:


(1.8) $$K_{k+1}=P_{k+1}\,H_{k+1}^{T}\,{\left(H_{k+1}\,P_{k\,1|k}\,H_{k+1}^{T}+R_{k+1}\right)}^{-1}$$

The state estimates at moment k+1 is:


(1.9) $${\hat{x}}_{k+1|k+1}={\hat{x}}_{k+1|1}+K_{k+1}(z_{k+1}-{\hat{z}}_{k+1|k}$$

State estimation error covariance matrix is:


(1.10) Pk+1∨k+1=(I-Kk+1⁢Hk+1)⁢Pk+1|k⁢(I-Kk+1⁢Hk+1)T+Kk+1⁢Rk+1⁢Kk+1T

Eqs. (1.2) to (1.10) form the extended Kalman filter algorithm.

In this paper, we define the failure state of GPS and VIO. When there is no GPS signal, it is defined as GPS failure. When the distance between the two adjacent VIO frames is greater than a given threshold, it is defined as the VIO failure. During the GPS or VIO fails, we perform sensor fusion by discarding the GPS or VIO state variable values, and replace the failed GPS or VIO values by the wheel odometer’s position, quaternion, and covariance values. Compared with the traditional Kalman filter algorithm, the proposed loosely coupled extended Kalman filter algorithm can perform tasks when the GPS or VIO fails, since the fusion mechanism includes the failure judgment of GPS and VIO. Therefore, the judgment of the fusion mechanism can remove the influence of the failed GPS or the invalid VIO on the whole system, and thus replace the state variables of a failed GPS or a failed VIO with the wheel odometer’s position, attitude, quaternion, and covariance values. Therefore, the system can still operate normally even when GPS or VIO fails.

The mathematical description of this paper is based on the extended Kalman filter theory, and the state variables involved are the position and attitude of GPS, wheel odometer, VIO, and the attitude of IMU. From a mathematical point of view, the proposed method is to switch the state variable to the value of the wheel odometer by judging the GPS signal state and the position change of VIO, in order to achieve system stability.

### Multi-Sensor Fusion

Multi-sensor fusion (MSF) currently completes the required measurement estimates for subsequent information processing by using computer technology. In this way, automatically analyze and synthesize data from multiple sensors or multiple sources with certain criteria.

The multi-sensor fusion is characterized by complexity and multi-level, and its basic requirements for algorithms are robustness, parallel processing capability, speed and accuracy of operations, etc. It is also necessary to consider the performance of the connection with the previous pre-processing (information input) and subsequent information processing system (system output), etc. In general, mathematical methods based on non-linearity and with features such as fault tolerance and adaptability can be used as fusion algorithms. At present, most of the research on sensor fusion algorithms based on Kalman filter include adaptive Kalman filter, extended Kalman filter, volumetric Kalman filter and unscented Kalman filter. In these studies, the model parameters and the system noise characteristics can be estimated and updated only when the sensor is working normally. When the sensor fails, the whole system collapse. In this paper, the failure judgment is added on the basis of the past, and a loosely coupled EKF algorithm is proposed to make the system run normally.

## Materials and Methods

### Algorithm Framework

**FIGURE 1:**
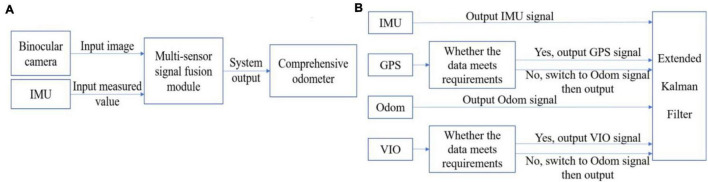
**(A)** Overall algorithm framework and **(B)** multi-sensor signal fusion module.

The proposed algorithm framework is shown in Figure 1. The input of algorithm are images captured by the binocular camera and the measurement from the IMU. And the information about the farming system, i.e, the farming data set, is obtained from the input binocular image. At the same time, the position pose estimation of the farming robot is completed based on the proposed fusion algorithm.

The extended Kalman filter fusion algorithm cannot obtain stable data since GPS and VIO are greatly affected by the environment. For example, GPS basically cannot receive satellite signals after being blocked, and VIO is too sensitive to ambient light. To solve this problem, the proposed algorithm fuses four sensors, among which the data from GPS and VIO need to be evaluated. The algorithm can perform decision-level fusion by adding a condition to determine whether the sensor fails. The algorithm judges whether the GPS is invalid through the differential state. When the output of the differential positioning state is 2, the GPS works normally. Otherwise, the algorithm will replace the GPS data with the ODOM data. The same goes for VIO. The algorithm determines whether the distance between the two adjacent VIO frames is greater than a given threshold. If the VIO fails, the proposed algorithm will use the ODOM data instead of the VIO data. When the sensor returns to normal, the data of GPS and VIO are re-added to the fusion system for fusion to correct the system error. After synchronizing the time of each sensor, the algorithm uses the EKF filtering formula to process the data, and outputs the attitude estimation value of the whole robot.

The data fused in the algorithm contain the odometer information converted by GPS through coordinates, including the covariance and coordinate values for x and y axes of the GPS; the covariance and coordinate values for *x* and *y* axes of the ODOM; the quaternion and covariance of the IMU; the covariance and coordinate values for *x*, *y*, and *z* axes of the VIO and the quaternion output by the tight coupling between the IMU and the camera. And all these data are obtained in the carrier coordinate system (*O_b_X_b_Y_b_Z_b_*). In addition, the coordinate systems considered in this paper include: Geographic Coordinate System (*O_g_X_g_Y_g_Z_g_*), Camera Coordinate System (*O_c_X_c_Y_c_Z_c_*), Navigation Coordinate System (*O_n_X_n_Y_n_Z_n_*), Inertial Coordinate System (*O_i_X_i_Y_i_Z_i_*) and Pixel Coordinate System (*O_p_uv*).

The proposed fusion of sensor information framework is shown in Figure 1, it can improve the navigation accuracy while enhancing the robustness of the system effectively. The data analysis and final results are given in the experiments and results section.

### Signal Fusion

In the signal fusion stage, we fuse the signals from IMU, ODOM, VIO, and GPS inputs based on the extended Kalman filtering algorithm in a loosely coupled manner. Then the fusion algorithm is used to estimate the real position and attitude information of the ground farm robot, and outputs the fused and filtered combined odometer (ODOM combined) information. The update of the positional attitude information, covariance information, and timing update are introduced as follows.

(1) Position-attitude update.
  All sensor sources have their own reference coordinate system and may drift with time. To solve this problem, we replace the absolute position pose information with the relative position pose difference.
(2) Covariance update.
  As the robot moves over a larger and larger range, the uncertainty of its position pose gradually increases, the covariance increases accordingly, and the absolute covariance of position pose become less meaningful; therefore, the sensors release a period of covariance change to update the covariance, i.e., the covariance of velocity.
(3) Timing update.
  It is assumed that the initial update moment of the farm robot to the filter is t_0. In this case, the filter subscribes to the fused position information at t_1, IMU information at t_2, ODOM information at t_3, GPS information at t_4, etc. Then the IMU, ODOM, and GPS information are interpolated at t_0 and t_1, t_0 and t_2, and t_0 and t_3, respectively. The EKF filter will use the information obtained from these linear interpolations to calculate the integrated odometer data updated by the filter at time t_1.

### Algorithm Input

**FIGURE 2:**
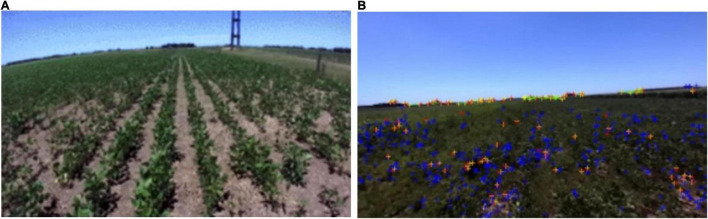
**(A)** Data set–repeated scenes and **(B)** example of dataset tracking.

**FIGURE 3:**
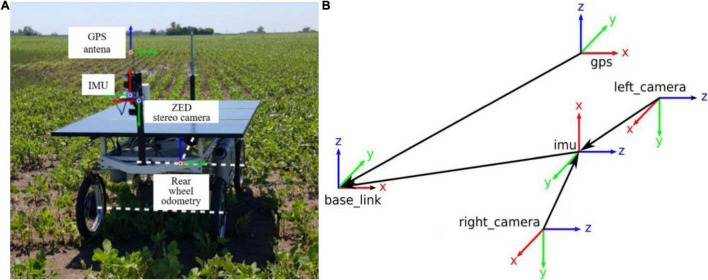
**(A)** Weeding mobile robot equipped with sensors and **(B)** dataset coordinate system definition.

The Rosario Dataset (Pire et al., 2019) is a class of publicly available datasets in agriculture (Figure 2), this dataset is used for mobile robots in agricultural scenarios in terms of agricultural sensor fusion, SLAM, etc. This dataset is provided by a weeding mobile robot equipped with a stereo camera, GPS-RTK sensor, and IMU (Figure 3A) for agricultural field. Figure 3B represents the dataset coordinate system, which means that the world coordinate system completes the pose transformation and turns to the map coordinate system. It is assumed that the initial update moment of the farm robot to the filter is t_0. In this case, the filter subscribes to the fused position information at t_1, IMU information at t_2, ODOM information at t_3, GPS information at t_4, etc. The collected information is fused with odometry, inertial, and visual information for further processing. Consequently, the results are generated by deriving environmental data covering highly repetitive scenes, reflection and burn images, direct sunlight scenes, and rough terrain scenes.

This dataset has a relatively universal character, including a range of different farmland scenes, and is suitable for the study of this paper. For this reason, the dataset is used as the initial input dataset for the proposed algorithm. In the dataset, Ground Truth is the real motion trajectory of the robot, i.e., the standard trajectory.

## Experimental Results and Analysis

In this paper, the Rosario Dataset is used to simulate the trajectory of the robot in the spatial Cartesian coordinate system by using the starting position of the robot as the zero point. All the data mentioned in the paper are obtained by running the proposed algorithm for the farming scenario.

Based on the unified dataset, the proposed approach analysis can be divided into the following three aspects.

(1) Judging the algorithm accuracy by comparing the trajectories of the fusion algorithm proposed in this paper with other algorithms.
(2) Judging the robustness of the proposed fusion algorithm based on the output trajectory by introducing Gaussian distribution noise to disable specific sensors.
(3) Changing the fusion judgment condition of VIO algorithm, output trajectory and observe the influence of different judgment conditions on the proposed fusion algorithm.

It should be noted that in the following simulation diagrams, the X direction represents the left and right transverse direction of the vehicle body, Y represents the front and rear longitudinal direction of the vehicle body.

### Experimental Simulation and Analysis for Trajectory Accuracy

#### Comparison of the Proposed Loosely Coupled Extended Kalman Filter Multi-Sensor Fusion Algorithm With the MSCKF_VIO Algorithm

**FIGURE 4:**
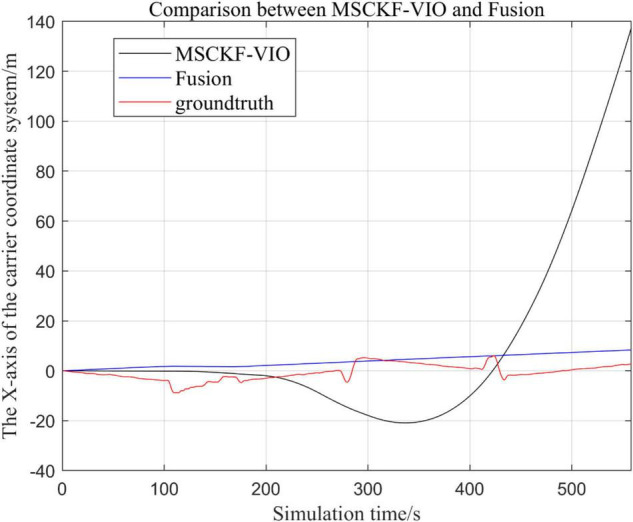
Comparison of the overall trajectory of the fusion algorithm with the MSCKF_VIO algorithm in the *x*-axis.

**FIGURE 5:**
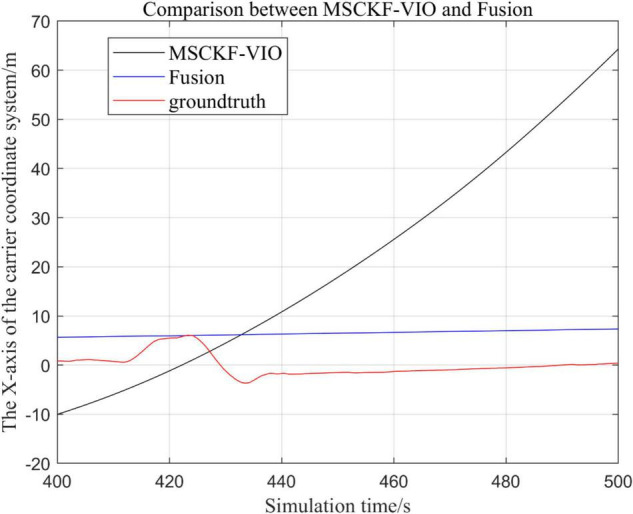
Comparison of the fusion algorithm with the MSCKF_VIO algorithm for zooming in on the *x*-axis local trajectory.

**FIGURE 6:**
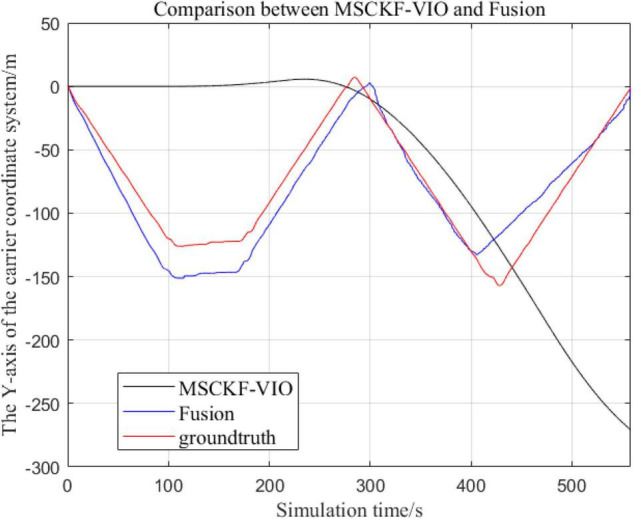
Comparison of the overall trajectory in *y*-axis between the fusion algorithm and the MSCKF_VIO algorithm.

**FIGURE 7:**
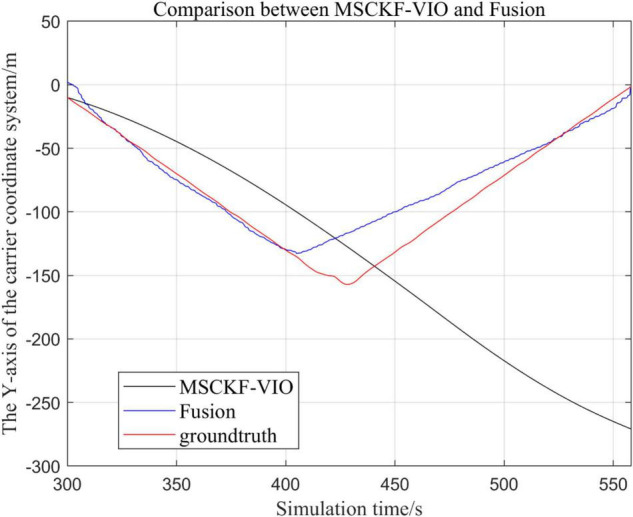
Zoomed-in comparison of the *y*-axis local trajectories of the fusion algorithm and the MSCKF_VIO algorithm.

**FIGURE 8:**
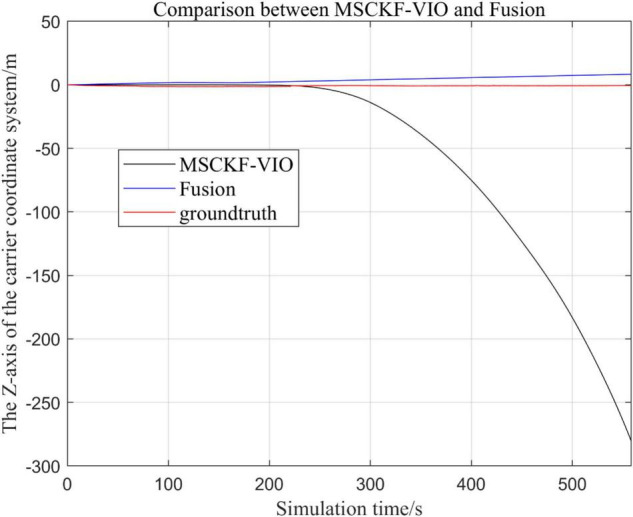
Comparison of the overall trajectory in *z*-axis between the fusion algorithm and MSCKF_VIO algorithm.

**FIGURE 9:**
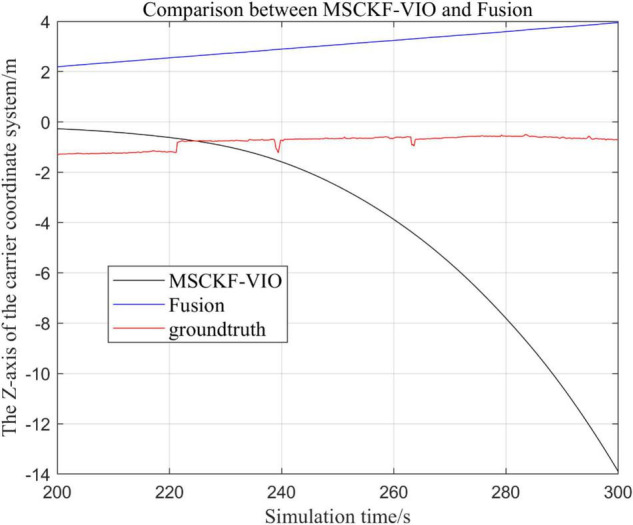
Zoomed-in comparison of the *z*-axis local trajectories of the fusion algorithm and the MSCKF_VIO algorithm.

There are three trajectories in the following figures. From Figures 4–9, the trajectory of loosely coupled extended Kalman filter algorithm is represented by Fusion, the trajectory of MSCKF_VIO is represented by MSCKF_VIO, and the standard trajectory is represented by ground truth, respectively. Where the Figures 5, 7, 9 intercept the part of *t*-axis coordinates from 0 to 150 s in Figures 4, 6, 8.

(1) Comparison of trajectories in the *x*-axis direction.
(2) Comparison of trajectories in the *y*-axis direction.
(3) Comparison of trajectories in the *z*-axis direction.
(4) Analysis of the figures.

By analyzing Figures 4–9, it can be found that when the robot first starts to run, the difference of the three trajectories is not large and the error is within the acceptable range. However, with the increase of running time, the trajectory error between the MSCKF_VIO algorithm and the standard trajectory is increasing. Furthermore, the speed of error increase is also improving. In contrast, the fusion algorithm proposed in this paper reflects its superior stability and higher accuracy in the overall trajectory.

#### Comparison of the Proposed Loosely Coupled Extended Kalman Filter Based Multi-Sensor Fusion Algorithm With Inertial Measurement Unit and ODOM Fusion Algorithm

**FIGURE 10:**
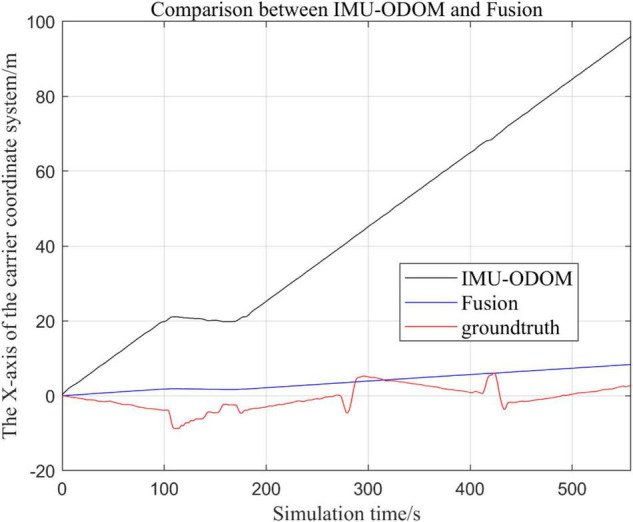
Overall trajectory comparison of fusion algorithm with IMU and ODOM fusion algorithm in *x*-axis.

**FIGURE 11:**
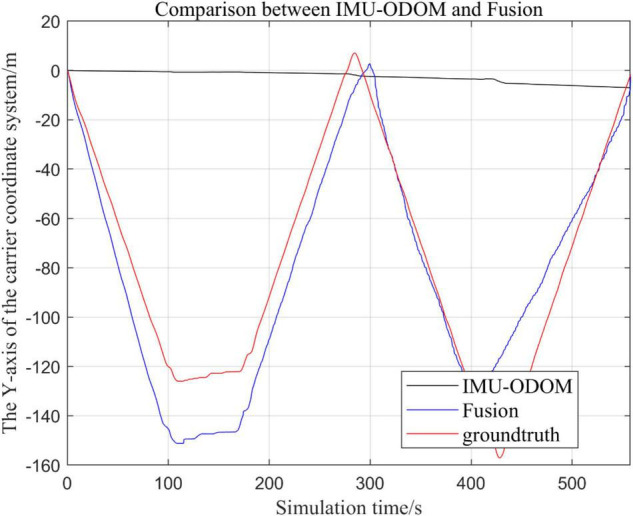
Overall trajectory comparison of fusion algorithm with IMU and ODOM fusion algorithm in *y*-axis.

**FIGURE 12:**
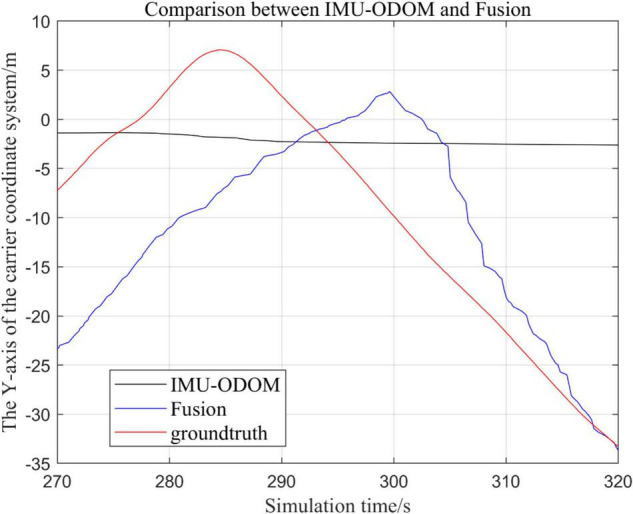
Comparison of *y*-axis local trajectory of fusion algorithm with IMU and ODOM fusion algorithm.

**FIGURE 13:**
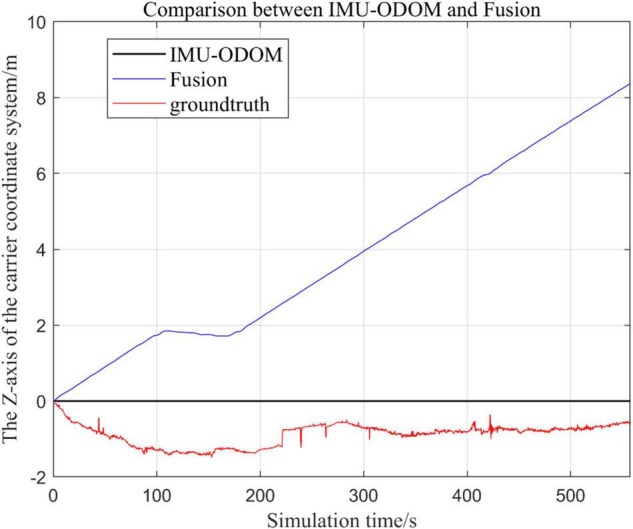
Overall trajectory in z-axis of fusion algorithm with IMU and ODOM fusion algorithm.

**FIGURE 14:**
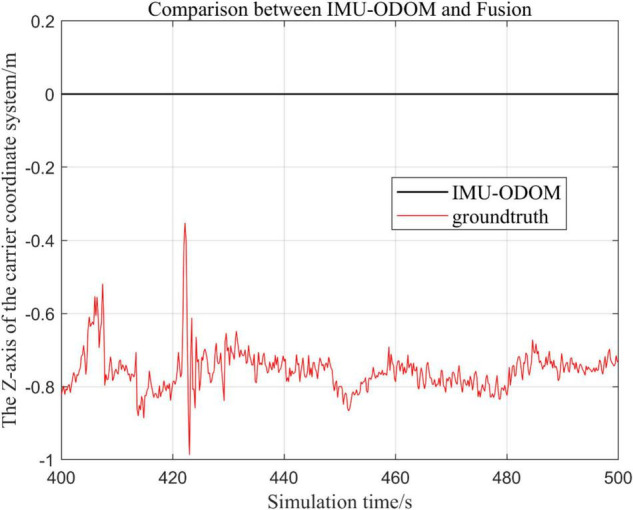
Comparison of *z*-axis local trajectory of fusion algorithm with IMU and ODOM fusion algorithm.

**FIGURE 15:**
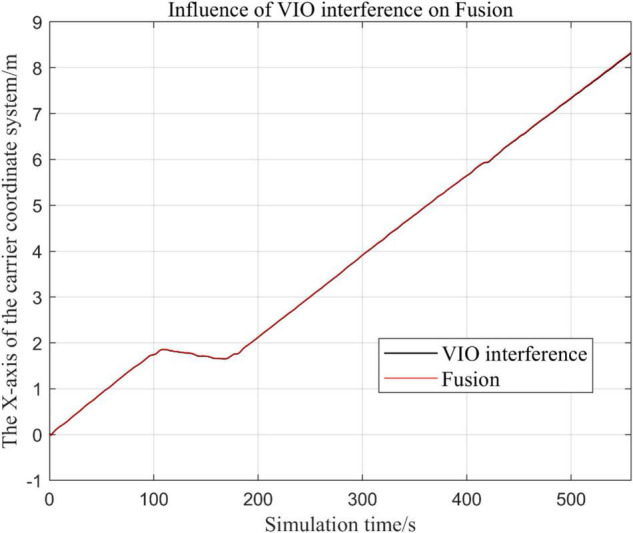
Comparison of the overall trajectory in the *x*-axis after VIO failure and when all sensors are working normally.

**FIGURE 16:**
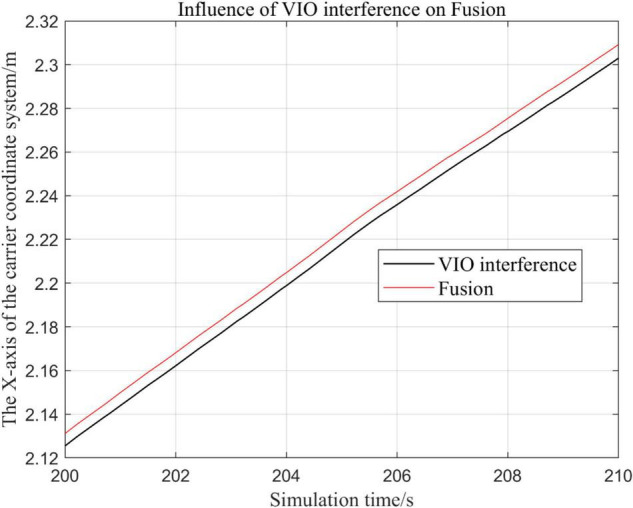
Comparison of local trajectory in *x*-axis after VIO failure and when all sensors are working normally.

There are three trajectories in the following figures. Figures 10–16 show the trajectory of the proposed loosely coupled EKF algorithm (denoted as Fusion), IMU-ODOM, and the standard trajectory (denoted as ground truth), respectively by IMU-ODOM. By comparing the trajectories of the Fusion algorithm, MSCKF_VIO algorithm and IMU and ODOM fusion algorithm proposed in this paper with the standard trajectories, the accuracy of the two algorithms can be judged.

(1) To make a more accurate analysis of the results, Figure 12 intercept the part of *t*-axis coordinates from 200 to 300 s in Figures 11, 14 remove the standard trajectory and intercept the part of *t*-axis coordinates from 400 to 500 s.
(2) Comparison of the trajectory in the *y*-axis direction.
(3) Comparison of trajectories in *z*-axis direction.
(4) Analysis of the figures.

By analyzing from Figures 10–13, in the *x*-axis trajectory, the accuracy of fusion algorithm of IMU and ODOM is obviously lower than the accuracy of the proposed fusion algorithm; in the *y*-axis trajectory, the difference in accuracy between the two is not significant. Since the car body moves in the plane, this paper only considers the *x* and *y* axes of the two-dimensional plane in the IMU-ODOM fusion simulation, and its value is zero compared to the real value in the z-axis trajectory.

#### Analysis of the Tables

In this paper, the results of different sensor fusion methods are quantitatively compared with standard trajectories, the average value and mean square deviation of the absolute value are shown in Tables 1, 2.

**TABLE 1:** Average of absolute value of errors in x, y, and z directions.

	Comparison		
Axis	Fusion-groundtruth	MSCKF-groundtruth	IMU-groundtruth
x/m	4.5948	20.9995	45.2532
y/m	14.9209	75.3966	75.0033
z/m	4.7763	54.2210	0.8808

**TABLE 2:** Mean square deviation of errors in x, y, and z directions.

	Comparison		
Axis	Fusion-groundtruth	MSCKF-groundtruth	IMU-groundtruth
x/m	2.6959	35.0502	25.8619
y/m	2.2211	96.6908	44.9099
z/m	2.2949	77.0708	0.2860

According to the conclusions drawn from the previous analysis with Tables 1, 2, it is concluded that the stability and robustness of proposed fusion algorithm are significantly superior to the MSCKF-VIO algorithm and the IMU-ODOM fusion algorithm, and it can be found that the trajectory of fusion is closer to the real trajectory than those obtained by other algorithms, and thus the accuracy of the proposed fusion algorithm is better than IMU and ODOM fusion algorithm.

### Experiments for the Robustness of the System

The robustness of the system reflects its characteristic of maintaining certain performance under certain parameter uptake. In this paper, the robustness of the system is verified by adding Gaussian distribution noise to disable a specific sensor, and then the trajectory with sensor disablement is compared to the trajectory without the disturbance.

In this part, we disable the sensors GPS and VIO respectively when t ∈ [200, 300].

### Visual Inertial Odometry Is Disturbed by Continuous Noise, While Other Sensors Operate Normally

**FIGURE 17:**
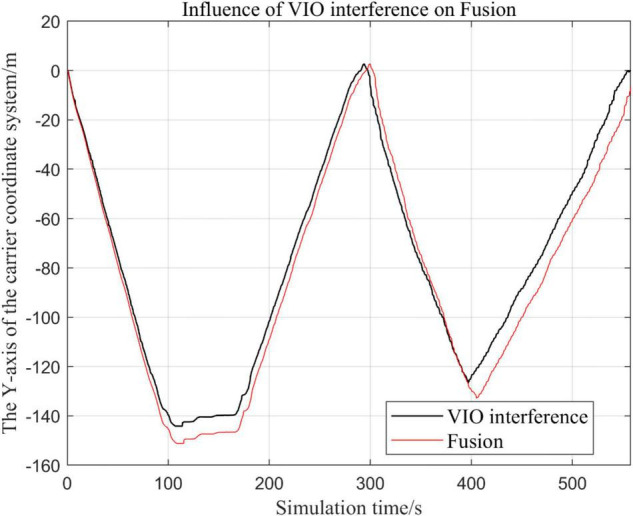
Comparison of the overall trajectory in *y*-axis after VIO failure and when the sensor is working normally.

**FIGURE 18:**
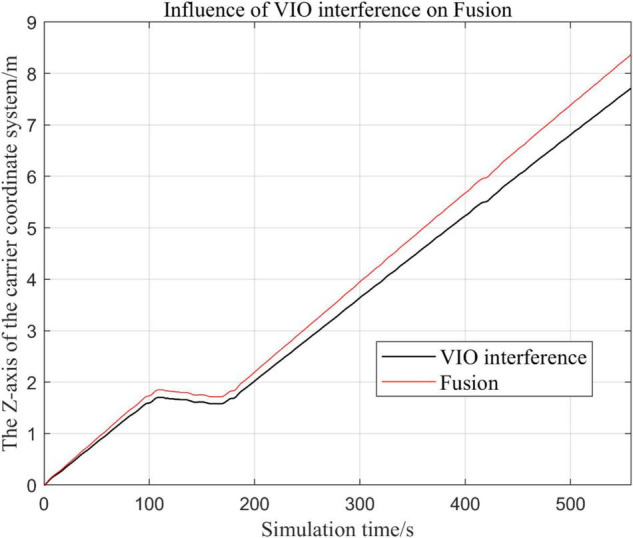
Comparison of the overall trajectory in *z*-axis after VIO failure and when all sensors are working normally.

In the following Figures 15–18, the Fusion trajectory represents the trajectory when all sensors are working normally, the VIO inference trajectory represents the trajectory when the VIO is disabled by noise interference, while Figure 16 is the trajectories for Figure 15 t ∈ [200, 210] and t ∈ [200, 300] sections and enlarged to allow a more accurate judgment of the results.

(1) Trajectory comparison in *x*-axis direction.
(2) Comparison of the trajectory in the *y*-axis direction.
(3) Comparison of trajectory in *z*-axis direction.

#### Global Positioning Navigation System Is Continuously Disturbed by Noise, Other Sensors Are Working Normally

**FIGURE 19:**
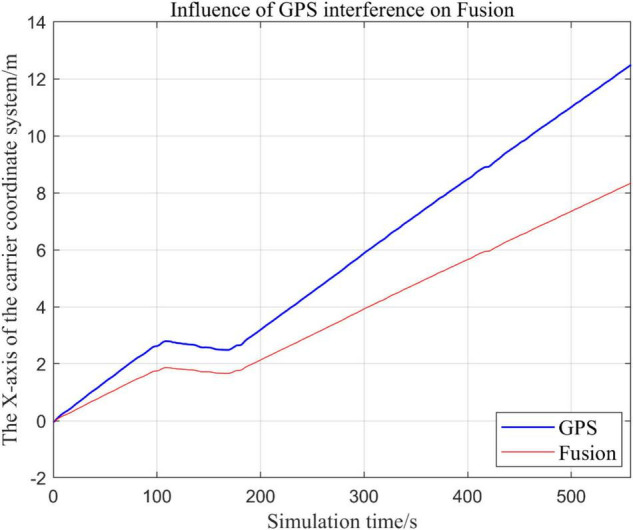
Comparison of overall trajectory in *x*-axis after GPS failure and when all sensors are working normally.

**FIGURE 20:**
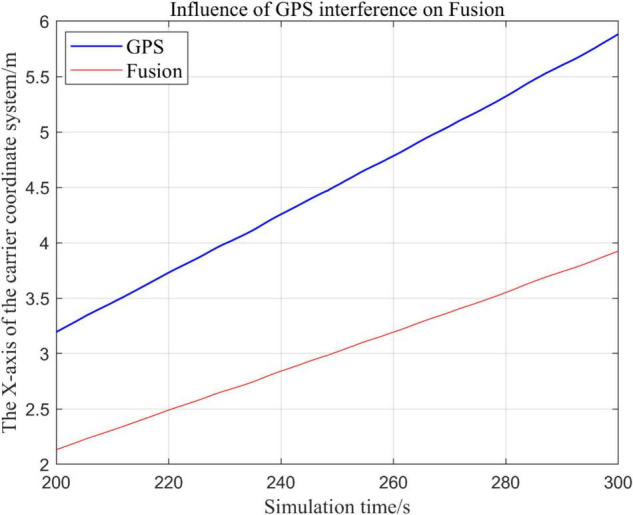
Comparison of the local trajectory in *x*-axis after GPS failure and when all sensors are working normally.

**FIGURE 21:**
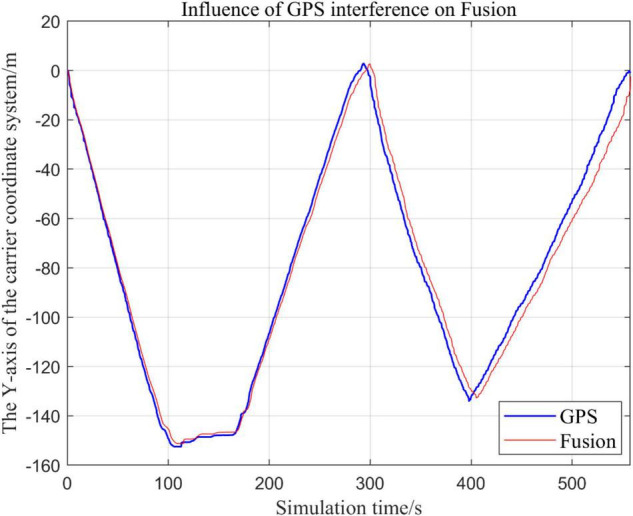
Comparison of the overall trajectory in *y*-axis after GPS failure and when the sensor is working normally.

**FIGURE 22:**
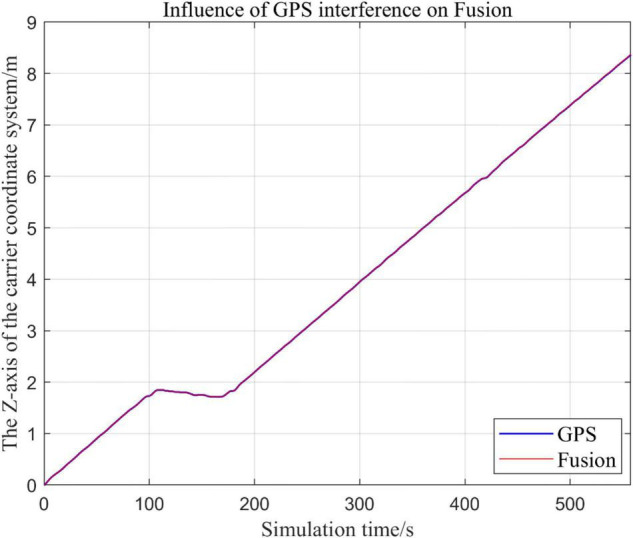
Comparison of the overall trajectory in *z*-axis after GPS failure and when the sensor is working normally.

From Figures 19–22, the Fusion trajectory represents the trajectory when all sensors are working normally, the GPS represents the trajectory when the GPS is disabled by interference, while Figure 20 are the trajectories of Figure 19 t ∈ [200, 300] sections and enlarged to allow a more accurate judgment of the results.

(1) Comparison of trajectory in *x*-axis direction.
(2) Comparison of the trajectory in the *y*-axis direction.
(3) Comparison of trajectory in *z*-axis direction.

#### Analysis of the Results

In this paper, we disable the sensors GPS and VIO respectively when t ∈ [210, 300], and enlarged the section of figure when t ∈ [200, 300]. In this way, we compare the two output trajectories and find that the robustness of the fusion algorithm proposed in this paper is better.

The above analysis of Figures 15–22 shows that the GPS or VIO failure does not have much effects on the proposed algorithm results, and it can be thus concluded that the proposed algorithm has excellent robustness ability to remain stable under a continuous disturbance.

### Effects of Different Judgment Conditions of Visual Inertial Odometry Algorithm Fusion on the System

#### Comparison of the Trajectories of the Fusion Algorithm Under Different Judgment Conditions

In the process of sensor fusion, this paper defines a threshold value for VIO sensors and sets judgment conditions based on this threshold value. If the VIO signal greater than this value, it is judged that the VIO data is not suitable for fusion, and the fusion algorithm of the VIO data is terminated.

In this paper, by changing the threshold value and observing the output trajectory, we study the influence of different thresholds judgment conditions on the trajectory results of the proposed fusion algorithm.

**FIGURE 23:**
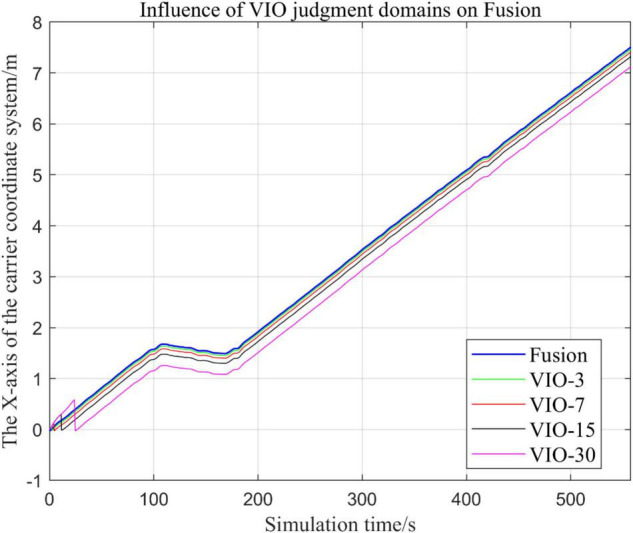
Comparison of the overall trajectory of the output *x*-axis with different thresholds.

**FIGURE 24:**
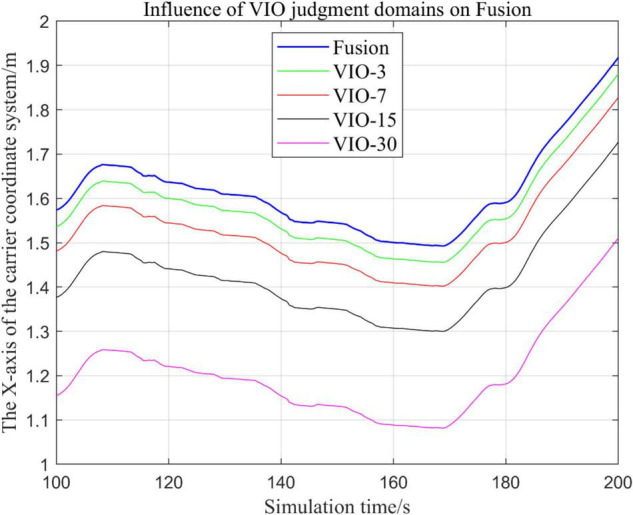
Comparison of output *x*-axis local trajectories under different thresholds.

**FIGURE 25:**
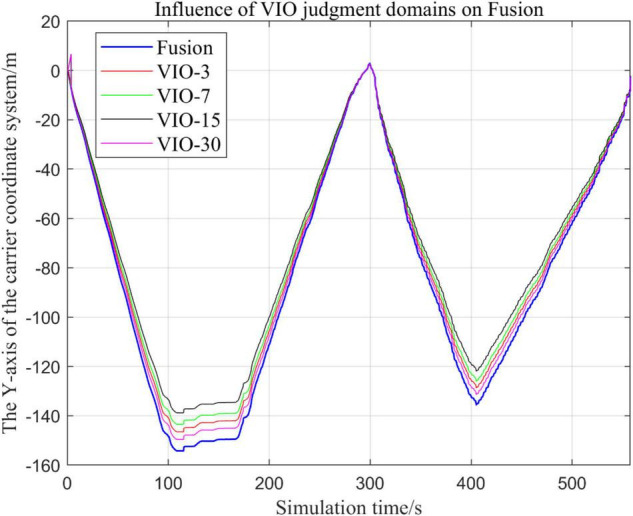
Comparison of the overall trajectory of the output *y*-axis under different thresholds.

**FIGURE 26:**
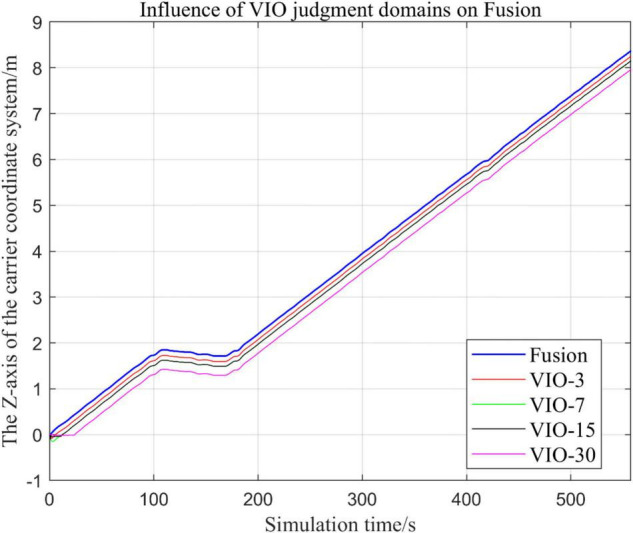
Comparison of the overall trajectory of the output *z*-axis under different thresholds.

**FIGURE 27:**
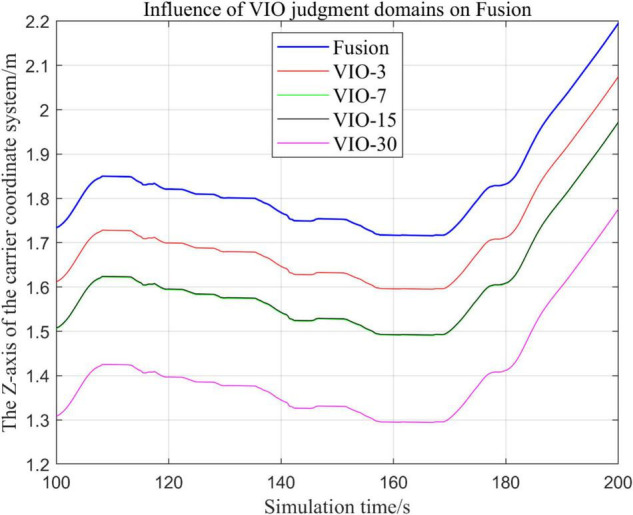
Comparison of output *z*-axis local trajectories under different thresholds.

In the following Figures 23–27, the Fusion trajectory represents the output trajectory when the threshold value is not changed, which is also the Fusion trajectory in all previous simulations with a threshold value of 0.3. VIO-3 represents the output trajectory with a threshold value of 3. VIO-7 represents the output trajectory with a threshold value of 7. VIO-15 represents the output trajectory with a threshold value of 15. And VIO-30 represents the output trajectory with a threshold of 30. Figures 24, 27 are the local trajectory results of t ∈ [100,200] sections from Figures 23, 26, respectively.

(1) Comparison of trajectories in the *x*-axis direction.
(2) Comparison of trajectories in the *y*-axis direction.
(3) Comparison of trajectories in *z*-axis direction.

#### Analysis of Results

In this paper, by changing the defined threshold value of VIO in the proposed algorithm and thus changing the fusion judgment condition, we observe the output trajectory results and analyze the effects of different thresholds on the overall algorithm outputs.

It can be seen from Figures 23–27 that, as the threshold value increases, the range of judging the VIO data to meet the fusion condition also increases. Based on the comparison between the output trajectories corresponding to different threshold values and the Fusion trajectories, it is observed that the trajectories on the *x*-axis and *z*-axis gradually approach the standard trajectories. It is thus inferred that the accuracy of the proposed fusion algorithm is improved with the increase of the threshold value within a certain range.

From the above experimental results, it can be concluded that the proposed multi-sensor fusion algorithm has a higher stability compared with traditional VIO algorithms such as MSCKF_VIO and the fusion algorithm of IMU and ODOM fusion algorithm. In addition, it also has excellent robustness. As the working time of the robot increases, the algorithm can still maintain a relatively stable trajectory, make up for the shortcomings of a single VIO, and thus solves the possible target loss and trajectory drift. It should be noted that, although the accuracy of the proposed algorithm has been greatly improved compared with the traditional VIO algorithm, there is still much room for accuracy improvement. Based on the relationship between the accuracy of the algorithm output trajectory and different threshold values, it is meaningful to find an optimal threshold values, in order to stabilize the trajectory errors in a small interval and make the algorithm output trajectory close to the standard trajectory.

## Conclusion

This paper proposes a loosely coupled EKF MSF algorithm for designing navigation systems. A series of experiments verified that the proposed algorithm has favorable robustness and stability against other methods. The proposed method provide reference significance for tasks such as navigation, localization, and path planning of agricultural robots.

In the future, we will establish a more extensive and complex dataset that are closer to practical applications, in order to further improve the robustness and accuracy of the algorithm under fast motion and more complex random scenarios. It is also interesting to find an optimal threshold values, in order to stabilize the trajectory errors in a small interval and make the algorithm output trajectory close to the standard trajectory.

## Data Availability Statement

Publicly available datasets were analyzed in this study. This data can be found here: http://www.cifasis-conicet.gov.ar/robot/.

## Author Contributions

ML and HW designed the research. ML and XF wrote the manuscript. ML, DZ, HW, XF, and WW conducted and analyzed the experiments. WW helped to edit the manuscript. DZ supervised the projected and helped to design the study. All authors contributed to the article and approved the submitted version.

## Conflict of Interest

The authors declare that the research was conducted in the absence of any commercial or financial relationships that could be construed as a potential conflict of interest.

## Publisher’s Note

All claims expressed in this article are solely those of the authors and do not necessarily represent those of their affiliated organizations, or those of the publisher, the editors and the reviewers. Any product that may be evaluated in this article, or claim that may be made by its manufacturer, is not guaranteed or endorsed by the publisher.
